# Cohort study of ageing from Bagé (SIGa-Bagé), Brazil: profile and methodology

**DOI:** 10.1186/s12889-021-11078-z

**Published:** 2021-06-07

**Authors:** Elaine Thumé, Marciane Kessler, Karla P. Machado, Bruno P. Nunes, Pamela M. Volz, Louriele S. Wachs, Mariangela U. Soares, Mirelle O. Saes, Suele M. Duro, Alitéia Santiago Dilélio, Luiz A. Facchini

**Affiliations:** 1 Post-Graduate Programme in Nursing, Federal University of Pelotas, Pelotas, Brazil; 2 Institute of Health Equity, University College London, London, UK; 3 Department of Social Medicine, Federal University of Pelotas, Pelotas, Brazil; 4 Post-Graduate Programme in Epidemiology, Federal University of Pelotas, Pelotas, Brazil; 5 Post-Graduate Programme in Health Science, Federal University of Rio Grande, Rio Grande, Brazil

**Keywords:** Cohort study, Epidemiology, Population based, Older adults, Health status, Health inequalities, Primary health care, SIGa-Bagé, Brazil

## Abstract

**Background:**

The Bagé Cohort Study of Ageing is a population-based cohort study that has recently completed the first follow-up of a representative sample of older adults from Bagé, a city with more than 100,000 inhabitants located in the state of Rio Grande do Sul, Brazil. This is one of the first longitudinal studies to assess the impact of primary health care coverage on health conditions and inequalities. Our aim is to investigate the prevalence, incidence and trends of risk factors, health behaviours, social relationships, non-communicable diseases, geriatric diseases and disorders, hospitalisation, self-perceived health, and all-cause and specific-cause mortality. In addition, we aim to evaluate socioeconomic and health inequalities and the impact of primary health care on the outcomes under study.

**Methods/design:**

The study covers participants aged 60 or over, selected by probabilistic (representative) sampling of the urban area of the city of Bagé, which is covered by Primary Health Care Services. The baseline examination included 1593 older adults and was conducted from July 2008 to November 2008. After eight to nine years (2016/2017), the first follow-up was conducted from September 2016 to August 2017. All participants underwent an extensive core assessment programme including structured interviews, questionnaires, cognitive testing (baseline and follow-up), physical examinations and anthropometric measurements (follow-up).

**Results:**

Of the original participants, 1395 (87.6%) were located for follow-up: 757 elderly individuals (47.5%) were re-interviewed, but losses in data transfer occurred for 22. The remaining 638 (40.1%) had died. In addition, we had 81 (5.1%) refusals and 117 (7.3%) losses. Among the 1373 older adults who were followed down, there was a higher proportion of female interviewees (p=0.042) and a higher proportion of male deaths (p=0.001) in 2016/2017. There were no differences in losses and refusals according to gender (p=0.102). There was a difference in average age between the interviewees (68.8 years; SD ±6.5) and non-interviewees (73.2 years; SD ±9.0) (p<0.001). Data are available at the Department of Social Medicine in Federal University of Pelotas, Rio Grande do Sul, Brazil, for any collaboration.

**Supplementary Information:**

The online version contains supplementary material available at (10.1186/s12889-021-11078-z).

## Introduction

Challenges related to ageing, such as the increased demand for health care and welfare [1], are a global concern. These issues are particularly pressing in middle- and low-income countries, which will be home to 80% of the population older than 60 years by 2050 [2].

Brazil has the fifth largest population in the world distributed over an extensive territory. Together with China and India, Brazil has slightly more than 20 years to adapt to an increase in the proportion of the population aged 60 years or older from 10% to 20%. France, for example, has approximately 150 years to adapt to this demographic change [2]. In Brazil, the proportion of people aged 60 years or older was 12.6% in 2017, and this value is projected to reach 29.6% by 2050 [3]. This change indicates a rapid ageing of the population in a context of limited resources and one of the largest levels of socioeconomic inequality (Gini coefficient = 0.53 in 2017) [4, 5].

Health status tends to be worse among older people, particularly in poorer countries [6, 7], where there is increased exposure to risk factors for chronic diseases [8] and premature morbidity and mortality [1, 9]. However, infectious/communicable diseases and external causes are still problems in low- and middle-income countries [1, 10].

In this context, data from longitudinal studies have the potential to support and evaluate short- and long-term social and health policies for elderly individuals [11]. Population-based longitudinal studies on this topic include a cohort study performed in Bambuí city, Minas Gerais, known as the Bambuí Cohort Study of Ageing [12]; a study in São Paulo known as the Study Health, Welfare and Aging (SABE) study [13]; the Epidemiology of the Older Adult (Epidoso) study [14]; an ageing cohort study in Florianópolis known as the EpiFloripa study [15]; and one recently for the entire country of Brazil, known as The Brazilian Longitudinal Study of Ageing (ELSI-Brazil) study) [11].

Nevertheless, the Bagé Cohort Study of Ageing (SIGa-Bagé Study) is the first population-based longitudinal study of elderly individuals aged 60 years or older to consider the association of health indicators in elderly individuals with primary health care (PHC) services. The study permits the evaluation of the prevalence, incidence and temporal trends of risk factors, health behaviours, social relationships, non-communicable diseases, geriatric diseases and disorders, self-perceived health and hospitalisation. It is possible to measure patterns of mortality, to determine its main causes, to measure survival and to identify temporal trends in socioeconomic inequalities and impacts on health. Finally, the study enables us to assess the influence of different models of health care - the traditional model and the Family Health Strategy (FHS) - on the outcomes under study and their ability to promote health equity, which sets this cohort apart from most others.

The FHS was decisive for the expansion and consolidation of PHC in Brazil. This programme was developed during the 1990s to reorganise and restructure the health system [16, 17]. The FHS has multidisciplinary teams, including community health workers, that are responsible for meeting the heath care needs of approximately 1000 households in a defined geographical area, bringing health care closer to where people live and work [17, 18]. The team delivers a range of services, including acute care, comprehensive and longitudinal healthcare, risk factor management, and referrals, and prioritises actions for health promotion, disease prevention and recognition of the health and social needs of the registered population and families [17, 18]. Professionals also deliver home health care for those who are unable to reach health services, such as those who are bedridden or have other serious health conditions [17]. Municipal governments are responsible for FHS service provision, but the majority of funding comes from the federal government.

FHS coverage is currently 63.9%, but is higher in rural areas and the poorest regions in order to reduce health inequalities [19]. The rest of the population has access to traditional primary health care (TPHC). TPHC teams do not have a fixed structure; contain more medical professionals, sometimes including specialists such as paediatricians, obstetricians and gynaecologists; do not serve a defined number of families or geographical area; and do not usually include community health workers [17]. TPHC focuses on specific diseases, dispenses curative care and acts on emerging demands, with little ability to solve health problems related to family and social issues [17].

Studies have shown that the FHS provides superior service performance compared with the traditional primary care model, particularly in terms of better quality of care [20, 21], reduced number of hospitalisations due to conditions sensitive to primary care [22, 23], reduced mortality due to cardiovascular disease [24] and increased access and use of health services and prevention, thus promoting health equity [17, 25].

The SIGa-Bagé baseline study was conducted in 2008, when the FHS covered 51% of the population in the urban area. Municipal management began to invest in the conversion of the traditional model to the FHS in 2003, and in the last 15 years, expansion with a consequent increase in access to health services has occurred, with 71.0% of the population now covered by the FHS [19].

## Methods/Design

### Where is it located and why?

The municipality of Bagé is located in the state of Rio Grande do Sul (RS), on the southern edge of Brazil; it had an estimated population of 121,143 inhabitants in 2019 and a demographic density of 28.52 inhabitants/km^2^ according to the 2010 Census [26]. The municipality is located just over 350 km from Porto Alegre (state capital) and approximately 190 km from Pelotas city, where the Federal University of Pelotas is located. It is bordered by Uruguay to the south and is the headquarters of the Seventh Regional Health Coordination. The main economic activity in the municipality is agriculture.

In 2010, the Municipal Human Development Index (MHDI) of Bagé was 0.740, which is considered high (MHDI between 0.700 and 0.799), similar to that of RS (HDI = 0.746) and of the entire country (HDI = 0.727). The dimension that most contributes to the municipal HDI is longevity (0.848), followed by income (0.739) and education (0.647) [27]. Bagé had Gross Domestic Product (GDP) of approximately R<DOLLAR/> 21930.77 per capita/year in 2016 [26].

Bagé municipality was chosen as the study area in 2008 based on the coverage rate of FHS (51.0%), which was the highest among all municipalities with over 100,000 inhabitants in the state of RS. In addition, the proportion of individuals aged 60 years or older (12.0%) was similar to the percentage found in RS (12.6%) but higher than that of the country as a whole (10.0%) [28].

### Who is included in the cohort?

The cohort consists of individuals aged 60 years or older living in private households in the urban area of Bagé municipality. In 2008, the sample size was calculated to study the “need for home care” and “home care received” [17, 29].

The prevalence of “need for home care” was calculated considering a population size of 14,792 elderly individuals in Bagé [28], a 95% confidence level, an estimated prevalence of the outcome of 20% and an acceptable error of 3%, resulting in a sample size of 653 elderly individuals. To calculate the prevalence of “home care received”, a population size of 3,000 elderly individuals with a need for home care, a 95% confidence level, an estimated prevalence of the outcome of 60% and an acceptable error of 4% were considered, resulting in a sample size of 483 elderly individuals. The calculation of associations considered a confidence level of 95%, a statistical power of 80%, 10% for losses and refusals, 15% for control of confounding factors and a design effect of 1.26 to detect relative risks of at least 1.5 exposures affecting up to 4% of the population. Thus, the total sample required for the study in 2008 was 1,530 individuals.

**Fig. 1:**
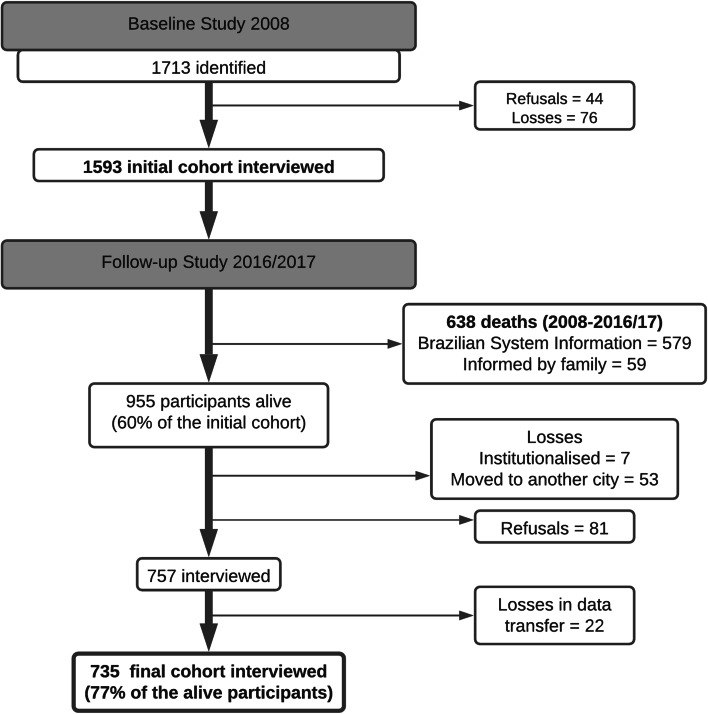
Sample recruitment process in the Bagé Cohort Study of Ageing, 2008-2016/2017. Pelotas, 2021

To locate the sample, basic health units were visited, and the teams were asked to demarcate the coverage areas on a map provided by the Brazilian Institute of Geography and Statistics (IBGE). These areas were divided into micro-areas: each block within the micro-area was numbered, and each quadrant of the block was identified for a subsequent draw of the starting point for data collection. After the draw, the household was visited, and the resident was asked about the presence of elderly individuals (60 years or older) in the household. One out of every six households was included, which allowed random identification of the individuals eligible for the study. No replacements were admitted. All elderly residents in the selected households were invited to participate in the study. Field workers made at least three attempts to interview household members. Ultimately, 1,713 elderly household members were identified, and a total of 1,593 were interviewed, for a response rate of 93% (Fig. 1). Refusals represented 3% (n=44) (FHS = 27.3% and traditional = 72.7%) and losses 4% (n=76) (FHS = 27.6% and traditional = 72.4%) of the identified household members [17, 29].

Losses and refusals were higher in wealthier areas of the city (traditional) and were mainly men, who often work all day, particularly in rural areas; some were travelling, and others had changed address or were hospitalised. However, considering the size of losses and refusals, no major effects are expected on the study’s estimates.

In 2016, follow-up of the individuals who had been interviewed in 2008 was conducted to establish a population-based longitudinal study. For both follow-ups, individuals who, at the time of the interview, were travelling, deprived of their liberty by judicial decision or living in long-term care facilities were excluded. Interviews not performed after three attempts on different days and times were considered losses/refusals, and no substitutions were allowed. In case of any cognitive or communication impairment of an elderly individual, the questionnaire was administered to the caregiver, excluding the questions about self-perception and self-assessment. During follow-up, the elderly individual was invited to answer the questionnaire with the possibility of rescheduling. For those not located, neighbours and health services were consulted regarding the current address or death of the elderly individual.

The list of deaths identified during the data collection was sent to the Health State Department for confirmation of the data and cause of death using the Mortality Information System, and a data use agreement was signed.

The structured questionnaire with pre-coded questions and the instruction manual were tested in a pilot study using individual interviews of elderly individuals in a long-term care facility. This strategy also enabled the practical training and selection of interviewers in July 2008 and September 2016. In the baseline study, the questionnaire was applied in paper format, whereas in the follow-up study, it was applied in an electronic format using a Mio P550B personal digital assistant (PDA).

Anthropometric measurements at follow-up were assessed based on the techniques proposed by Lohman [30]. Weight was measured on a Tanita *Ⓡ* digital scale, model UM-080, with a maximum capacity of 150 kg and a precision of 100 g; elderly individuals were weighed wearing light clothing and while barefoot. Knee height was obtained using an Indaiá *Ⓡ* wooden stadiometer for children, with a 100-centimetre scale graduated in millimetres; for this purpose, the individual was asked to remain seated while barefoot and to keep the knee flexed at a 90^∘^ angle.

Waist circumference (WC) and calf circumference (CC) were measured using an inextensible anthropometric tape. For WC, the interviewee faced the examiner while standing and lifted their shirt up to the lower edge of the nipples; the tape was then placed around the waist of the individual at the midpoint between the lower edge of the last rib and the iliac crest with 0.1-cm precision. CC was measured at the maximum horizontal distance around the calf. To measure grip strength, a JAMAR *Ⓡ* device with a precision of 150 cm was used. The elderly individual was seated with the arm close to the body and in a 90^∘^ flexed position, and grip movement was measured three times for each hand.

Blood pressure was measured using an Omron *Ⓡ* device, model HEM-6123, and the measurement was standardised on the left arm. The first measurement was taken 15 minutes after beginning the interview, and two other measurements were taken at the end of the questionnaire. All devices used for anthropometric measurements were registered at the National Institute of Metrology, Quality and Technology (INMETRO).

The study was advertised in the municipality through radio interviews, articles in local newspapers and the creation of a page on a social network site to reduce the number of refusals and losses.

Ethical principles were ensured, and the interviewees or their guardians were asked to sign an informed consent form. The right not to participate in the study and the anonymity of the interviewees were ensured. This cohort study project was submitted to the Brazil Platform and approved by the Research Ethics Committee at Federal University of Pelotas under number 678,664 and was granted a Certificate of Ethical Appreciation under number 31497314000005317.

### Cohort waves: what has been measured?

To date, two data collections have been performed: the first was conducted from July to November 2008 (baseline), and the second was conducted from September 2016 to August 2017 (follow-up).

In 2008, data were collected about social and health conditions and utilisation of health care services, including socioeconomic, demographic, behavioural, home care, morbidity, functional capacity, self-perception of health, support network, feeding guidelines, physical activity and anthropometric (self-reported) data. The following validated instruments were used in both collections: the International Physical Activity Questionnaire (IPAQ SHORT) [31], the Katz Index of Independence in Activities of Daily Living (Katz ALD) [32], the Lawton Instrumental Activities of Daily Living Scale (IADL) [33], the Geriatric Depression Scale (GDS) [34], and the Mini Mental State Examination (MMSE) [35]. The following instruments were included in the follow-up: the Quality of Life Assessment (WHOQOL-BREF) [36], the Patient Health Questionnaire (PHQ-9) [37], the Patient Assessment of Chronic Illness Care (PACIC) [38], the Vulnerability to Abuse Screening Scale (VASS) [39], the Medical Outcomes Study’s Social Support Scale [40], and other questions developed specifically for the study (Additional file 1: Questionnaire).

The follow-up was planned to collect the same baseline data and some new variables, including complementary instruments and/or questions related to support and social support, stressful events, risk of abuse, quality of life and anthropometric measurements.

Among the interviews conducted, 10% were randomly selected for quality control via telephone. For that purpose, 14 key questions were selected from the original questionnaire, in addition to four questions to be completed by the researcher. This approach allowed the identification of possible errors and/or fabricated responses, with the objective of evaluating intra-observer reliability. The phone calls were made within two weeks of the interview.

## Results

Of the 1593 elderly individuals in the baseline study, 1395 (87.6%) were located for follow-up: 757 elderly individuals (47.5%) were re-interviewed (FHS = 54.7% and traditional = 45.3%), with 22 losses in data transfer; 579 deaths were confirmed by the Brazilian System of Information on Mortality (SIM) until August 2017, and 59 deaths were reported by the family or neighbours but were not located in the SIM, giving a total of 638 deaths (40.1%) (FHS = 53.4% and traditional = 46.6%). Among the participants in 2008, 198 (12.4%) were not followed up in 2016-2017 FHS= 49.5% and traditional = 50.5%): 81 (5.1%) were refusals, and 117 (7.3%) were classified as losses. Among the losses, 57 could not be located, seven were institutionalised, and 53 had moved to another municipality (Fig. 1).

Among the 1373 older adults followed up in 2016/2017 (735 interviews [53.5%] + 638 deaths [46.5%]), there was a higher proportion of female interviewees (48.1% females and 42.8% males, p = 0.042) and a higher proportion of male deaths (45.2% males and 37.0% females, p = 0.001). There were no differences in losses and refusals according to gender (14.9% women and 12.0% men, p = 0.102). There was a difference in average age between the interviewees (68.8 years; SD ±6.5) and non-interviewees (73.2 years; SD ±9.0), with a *p*-value ¡ 0.001. The overall mortality rate was 72.3 (66.9-78.1) deaths per 1000 person-years over the course of a mean duration of follow-up of 6.4 years (SD: 2.6). Cause of death was ascertained for 579 deaths, and the main specific causes were cardiovascular diseases (36.9%), cancer (23.8%) and respiratory diseases (12.2%).

Table 1 shows the demographic, socioeconomic and health behaviours distribution among older adults in the baseline study (2008) and in the follow-up (2016/2017).

**Table 1:** Demographic, socioeconomic and behavioural characteristics at baseline, re-interview, deaths and losses. SIGa-Bagé Cohort, 2008-2016/2017

	Baseline 2008		Re-interview		Deaths		Losses and	
Variables	Cohort 2008		2016/2017		2008-2017		Refusals 2017	
	(n=1593)		(n=735)		(n=638)		(n=220)	
	n	%	n	%	n	%	n	%
**Gender**								
Male	593	37.2	254	34.6	268	42.0	71	32.3
Female	1000	62.8	481	65.4	370	58.0	149	67.7
**Age (2008)**								
60 to 64 years	400	25.1	-	-	99	15.5	75	31.5
65 to 74 years	696	43.7	309	42.0	223	35.0	106	48.2
75 years or older	497	31.2	426	58.0	316	49.5	39	17.7
**Self-reported colour**								
White	1252	78.6	604	82.2	501	78.5	174	79.1
Black/Brown/Indigenous/Yellow	341	21.4	131	17.8	137	21.5	46	20.9
**With partner**	816	51.3	310	42.2	292	45.8	124	56.6
**Live alone**	280	17.6	177	24.1	113	17.7	41	18.6
**Retiree**	1142	71.7	584	79.7	471	73.8	151	68.6
**Education level**								
Iliterate	382	24.0	175	27.5	175	27.5	54	24.6
1 to 7 years	868	54.5	348	54.6	348	54.6	107	48.6
8 years	342	21.5	114	17.9	114	17.9	59	26.8
Economic classification*								
A/B (Richest)	429	27.1	105	14.6	138	21.9	73	33.5
C	615	38.9	283	39.3	256	40.6	82	37.6
D/E (Poorest)	537	34.0	332	46.1	236	37.5	63	28.9
**Smokers**	244	15.3	68	9.3	97	16.8	40	14.4
**Alcohol consumption**	254	16.0	105	14.4	61	10.6	45	16.3

* Brazilian Economic Classification Criteria.

Based on the data obtained from the 2008 data collection, 62.8% of the elderly individuals were female, 51.3% were married or lived with a partner, 71.7% were retired, 17.6% lived alone, and a high proportion had no schooling (24.0%) and were poor (34.0%). The 2008 sample has several similarities to the Bagé city profile but is slightly older and female. There is no information available on individual socioeconomic status in the city profile, but skin colour is a useful indicator [26]. In 2016/2017, most of the interviewees were female (65.4%), 42.2% were married or lived with a partner, 79.7% were retired, and 24.1% lived alone. The proportion of older adults with no schooling (23.2%) was similar to that in 2008, whereas the proportion of poor older adults (46.1%) was higher.

Among the main results published from the baseline study (2008), 34.1% of the participants were assessed with cognitive impairment [41], 18.0% had depressive symptoms [42], 28.0% reported falls in the year prior to the interview [43], 10.6% had functional incapacity for Basic Activities of Daily Living, 34.2% had functional incapacity for Instrumental Activities of Daily Living [44], 37.4% reported spinal disorders (91.5% for more than 12 months) [45], and 20.7% reported urinary incontinence [46]. The prevalence of multimorbidity was 81.3% for ≥2 morbidities and 64.0% for ≥3 morbidities [47]. In addition, 51.0% of the participants with hypertension and/or diabetes had weak informal relationships (little contact with friends, neighbours and relatives) [48], 69% of the elderly individuals reported having pets [49], and 32.9% of the elderly individuals who used medication were classified as having low adherence to treatment [50]. The rate of home health care utilisation was 7% but was significantly higher in areas with FHS coverage compared to areas covered by TPHC [17]. Nursing staff provided most home care within the FHS, and two-thirds of the elderly individuals who requested home care reported improved health status [29]. Overall, 17.7% had been hospitalised, and 10.6% had experienced non-surgical hospitalisation in the last year prior to the interview [51]. The vast majority of these papers presented social and health inequalities.

To date, four theses (about the incidence of depression, association between social relationships and mortality, quality of health care for chronic conditions, mortality in older adults and the role of the FHS in promoting health equity) and one dissertation (occurrence of urinary incontinence) have been finalised using data from the cohort study. In addition, three further theses and one dissertation are in progress that address nutritional status and mortality; the incidence of functional disability; falls and mortality; and recently, coronavirus disease 2019 (COVID-19).

Two papers were recently published, and one was accepted; these papers report results from the cohort study and longitudinal analysis [52–54]. In a study comparing SIGa-Bagé and The English Longitudinal Study of Ageing, Kessler et al. [52] verified that mortality rates were higher in SIGa-Bagé, with physical inactivity and current smoking having the strongest association in both cohorts. A clear graded relationship between the number of risk factors and subsequent mortality was found, with wealth gradients of mortality in both cohorts [52]. In a study of social relationships and survival, Soares et al. [53] found that older adults who went out of their homes daily had considerably lower mortality and that going to parties had a protective effect for survival. The lower risk of death for women was modified when the older adults lived in households with two or more people [53]. Another study from Kessler et al. [54] identified that the FHS modified the effect of wealth on mortality, reducing social inequalities in mortality among older adults [54].

## Discussion

The SIGa-Bagé Study is a longitudinal, population-based study with a low probability of selection bias due to probabilistic sampling and population distribution in the territory. It was expected that the female population represented 59% of the total population with 60 years or older. However, our study enrolled 62.3% of women, showing a slight female over representation in the cohort. In addition, the nine-year follow-up period and low number of losses and refusals are notable. This is one of the first Brazilian longitudinal studies on ageing with an emphasis on PHC that aims to identify differences in the exposure of health indicators and the impact of health services. The instruments and methods used were consistent with those of the first collection performed, ensuring comparability for most outcomes. New technologies were implemented for data collection, and new thematic areas were added to the study.

The characterisation of the cohort regarding social, psychological and biomedical dimensions, use of health services and mortality will allow the analysis of social gradients in health and mortality and the capacity of health services, especially those of PHC, to promote health equity in such an uneven scenario. In addition, we will be able to recognise mediating mechanisms. The social diversity captured in the cohort will allow an in-depth investigation of health inequalities. These results will be particularly important given the scarcity of investments in health and the scarcity of high-quality and strong evidence on the impact of public health policies in low- and middle-income countries. Results such as these will empower Brazilian’s PHC and strengthen the Unified Health System (SUS).

This study also has limitations. The study began by selecting participants aged 60 years or older, which prevents an analysis of the transition of health status from younger to older adults. We did not conduct cohort replacement in 2016/2017 due to the importance of updating the sample to maintain an adequate proportion of participants at the lower end of the age group and facilitate comparison between cohorts. Due to budget constraints, the first follow-up was carried out only after a period of 8-9 years, so the changes that occurred during this time interval were not monitored. Another limitation is the long period of data collection; due to budget constraints, we were not able to hire enough interviewers to collect the data more quickly. Previous personal history information was collected retrospectively; however, cohort studies typically measure exposures before the onset of disease or outcome to avoid reverse causality. Selection and memory biases, which generally affect case and control studies, are also less frequent[55].

Although the results of this study cannot be extrapolated for the country due to socioeconomic and cultural differences by regions, longitudinal studies carried out in other Brazilian regions, states [12–15] and in the country [11] have reported profiles of older adults similar to that in the SIGa-Bagé Study. In this sense, the results can support public policies and health care aimed at promoting healthy ageing and health equity at the local, regional and national levels. Moreover, the results can be used to support permanent education programmes and professional training to improve the quality of health care delivery, especially in PHC services.

## Data access and collaboration

We invite interested parties from national and international institutions to learn more about our cohort and form partnerships and collaborations to build research networks and strengthen skills and capabilities among researchers. Our aim is to build an international research network with a special focus on health inequalities and the role of PHC in promoting health equity. Currently, there is a partnership in place with the Research Department of Epidemiology and Public Health at University College London, England (UK), and the Global Health Institute at Duke University, United States (USA). Analyses and articles are in progress and will be published later in leading international journals.

## Supplementary Information

**Additional file 1** Questionnaire.

## Data Availability

More information on the SIGa-Bagé study and materials can be found on the AQUARES group website: Access and Quality of the Health Care Network https://dms.ufpel.edu.br/aquares/assistencia-domiciliar-a-idosos/ or upon request by contacting Professor Dr Elaine Thumé by email: elainethume@ufpel.edu.br

## Abbreviations

SIGa-Bagé Study: Bagé(Brazil) Cohort Study of Ageing
PHC: primary health care
FHS: Family Health Strategy
SUS: Brazilian Unified Health System
COVID: coronavirus disease
SIM: Mortality Information System
CI: confidence interval. IPAQ: International Physical Activity Questionnaire
ALD: Activities of Daily Living
IADL: Instrumental Activities of Daily Living Scale
GDS: Geriatric Depression Scale
MMSE: Mini Mental State Examination
WHOQOL: Quality of Life Assessment
PHQ: Patient Health Questionnaire
PACIC: Patient Assessment of Chronic Illness Care
VASS: Vulnerability to Abuse Screening Scale
WC: waist circumference
CC: calf circumference
IBGE: Brazilian Institute of Geography and Statistics
PDA: personal digital assistant
RS: Rio Grande do Sul.
