# Supplementary material for: Cohort study of ageing from Bagé (SIGa-Bagé), Brazil: profile and methodology
Source: BMC Public Health. 2021 Jun 7;21:1089. doi: 10.1186/s12889-021-11078-z (PMC8182998; doi:10.1186/s12889-021-11078-z)
Supplement: Supplementary file 1 — Additional file 1 Questionnaire. [file 12889_2021_11078_MOESM1_ESM.pdf]

Federal University of Pelotas<br>Posgraduate Program in Epidemiology<br>Questionnaire – Elderly – 60 years old or more	Do not write in this column
**IDENTIFICATION**	
Interviewer number: __ __	*NUENT__ __*
Basic health Unit: __ __	*UBS __ __*
Micro-area number: __ __	*MICRO __ __*
Block number: __ __	*QUADRA __ __*
House number: __ __ __	*DOM __ __ __*
Number of person in the household: __ __	*NPED__ __*
Interview date: __ __/ __ __	
Interview start time: __ __ : __ __ hs	
Address____	
Contact phone: (___) ________________________	
**ATTENTION INTERVIEWER: DO NOT ASK, JUST OBSERVE**	
**1.** Skin color or race of the interviewee:<br>(1) White (2) Black (3) Yellow (4) Brown (5) Indigenous	*CORPEL __*
**2.** Gender of the interviewee:<br>(0) Male (1) Female	*SEXO __*
**START INTERVIEW: INTERVIEWEE IDENTIFICATION DATA**	
**3. What is your name?**	
**4. How old are you?** __ __ __ (full years)	*IDADE __ __ __*
**5. What is your date of birth?** _ _ / _ _ / _ _ _ _	DN__ __ __ __ __ __ __ __
**ASK THE INTERVIEWEE**	
**6. What is your current weight?** __ __ __ , __ kg<br>(888,8)NSA* (999,9)IGN**<br>*NSA= Does Not Aplied ** IGN= Ignored	*PEK__ __ __,__*
**7. How tall are you?** __ __ __ cm (888)NSA (999)IGN	*ALTC __ __ __*
**8. What color is your skin or race?**<br>**(1) White (2) Black (3) Yellow (4) Brown (5) Indigenous (9) IGN**	*CORAUT __*
**9. In your opinion, what color is my skin or race?**<br>**(1) White (2) Black (3) Yellow (4) Brown (5) Indigenous (9) IGN**	*CORENT __*
**10. Did you attend school?**<br>(0) No - SKIP TO QUESTION 12<br>(1) Yes (9)IGN	*FREQESC __*
**11. Up to which grade did you study?**<br>Series: ______________________________Degree:___________________<br>(Code after closing the questionnaire)<br>Complete years of study: __ __ years (88)NSA (9)IGN	*SERESTA__ __*
**12. Do you know how to read and write?**<br>(0) No<br>(1) Yes<br>(2) Only sign (9) IGN	*LERESC __*
**13. Did you work, being paid, in the last month?**	*TRABULTM __*

(0) No<br>(1) Yes (9)IGN	
**14. Are you retired?**<br>(0) No - SKIP TO QUESTION 16<br>(1) Yes (9)IGN	*APOS* __
**15. At what age did you retire**? __ __ Years old<br>(88)NSA (99)IGN	*IDAPOS* __ __
**16. What is your current marital status**<br>(1) Married<br>(2(Single or without a partner - SKIP TO QUESTION 18<br>(3) Separated or divorced - - SKIP TO QUESTION 18<br>(4) Widowed - - JUMP FOR QUESTION 18	*SITCONJ*__
**17. How old is your spouse / partner?**<br>__ __ __ (complete years) (888)NSA (999)IGN	*IDESPA* __ __ _
**18. Do you have or have had children (includes adopted children)?**<br>(0) No - SKIP TO QUESTION 20 (1) Yes (99)IGN	*FILHO*__
**19. If YES, how many?**<br>__ __ (number of male children)<br>__ __ (number of female daughters) (88)NSA (99)IGN	*FILQTH*__<br>*FILQTM* __
**20. The house in which you live is:**<br>**(0) Own**<br>**(1) Rented**<br>**(2) From a relative or friend. Which one. Which?**______________________<br>(9)IGN	*CASAPR*__<br>*CASAPRQ*__
**21. Do you live alone?**<br>(0) No (1) Yes - SKIP TO QUESTION 24	*MORSOZ*__
**22. In addition to you, how many people live in this house?**<br>__ __ people (88)NSA	*QTSMOR*__ __
**23. What is the relationship of these people with you?**<br>Spouse / partner (0) No (1) Yes (8) NSA<br>Father (0) No (1) Yes (8) NSA<br>Mother (0) No (1) Yes (8) NSA<br>Grandson (0) No (1) Yes (8) NSA<br>Father-in-law/Mother-in-law (0) No (1) Yes (8) NSA<br>Children / daughter (0) No (1) Yes (8) NSA<br>Brother / sister (0) No (1) Yes (8) NSA<br>Other family members (0) No (1) Yes (8) NSA<br>Employee (0) No (1) Yes (8) NSA<br>OtherEmployee: ___________________________	*ESPMO*__<br>*PAIMO* __<br>*MAEMO*__<br>*NETOMO*__<br>*SOGMO*__<br>*FILHOMO*__<br>*IRMOR*__<br>*OUTMO*__<br>*EMPMO*__<br>*OUMO*__
**24. Do you usually be alone during the day (day and night)?**<br>(0) Never or rarely<br>(1) Yes, about an hour<br>(2) Yes, long periods of time - ex: every morning, every afternoon<br>(3) Yes, only during the day<br>(4) Yes, only at night<br>(5) Yes, alone all the time (9) IGN	*FICARSOZ* __
**25. Do you use any of this equipment or accessories in your daily life?**<br>**Bengal** (0) No (1) Yes (8) NSA<br>**Walker** (0) No (1) Yes (8) NSA<br>**Wheelchair** (0) No (1) Yes (8) NSA<br>**Hearing aid (in ear)** (0) No (1) Yes (8) NSA<br>**Upper denture** (0) No (1) Yes (8) NSA<br>**Denture at the bottom** (0) No (1) Yes (8) NSA<br>**Femur prosthesis** (0) No (1) Yes (8) NSA<br>**Foam mattress with dots (pyramidal)** (0) No (1) Yes (8) NSA<br>**Air cushion for chair or bed** (0) No (1) Yes (8) NSA<br>**Others:**___________________________________	*USABENG*__<br>*USAAND*__<br>*USACADR*__<br>*USAAPARAUD*__<br>*USADENTSUP*__<br>*USADENTINF*__<br>*USAPROTFEM*__<br>*USACOP* __<br>*USAALM* __<br>*USAOUT*__
**26. How do you consider your health?**<br>*SHOW THE FACES CARD!*<br>**(1) Poor (2) Bad (3) Regular (4) Good (5) Great (9) IGN**	*ISAUD* __
**27. Compared to <THE LAST 5 YEARS>, you would say that your health today is:**<br>**((1) Better (2) Same thing (3) Worse** (9)IGN	*ISAUDH* __

**28. In comparison with other people your age, would you say that your health is:**<br>**(1) Better (2) Equal (3) Worse** (9)IGN	*ISAUOUT__*
**29. How do you feel about your life in general?**<br>**(0) Dissatisfied**<br>**(1) Satisfied - SKIP to question 31** (9)IGN	*ISENT __*
**30. What are the main reasons for your dissatisfaction with life? (Note up to 3 reasons**<br>**(1) Economic problem (debt, little money);**<br>**(2) Health problem;**<br>**(3) Housing problem;**<br>**(4) Transport problem (there is no way to leave the house);**<br>**(5) Conflict in personal relationships;**<br>**(6) Lack of activity**<br>**(7) Another problem**____________________________________ (8)NSA (9)IGN	*IMDIMP1__*<br>*IMDIMP2__*<br>*IMDIMP3__*
**31. Since <THREE MONTHS AGO> did you consult with any doctor, in an emergency service (SAMU, Emergency Room)?**<br>(0) No<br>(1) Yes, how many___ ___times (99)IGN	*CONM3__ __*
**32. Since <THREE MONTHS AGO> did you consult with a doctor in services that were not urgent?**<br>(0) No - SKIP TO QUESTION 34<br>(1) Yes, how many___ ___times (99)IGN	*CONMA__ __*<br>*CONNUVEZ__ __*
**33. IF YES, where was your last appointment?**<br>**(01) Health post**<br>**(02) Private doctor**<br>**(03) Doctor with health agency**<br>**(04) Other**__________________________ (88)NSA<br>(99)IGN	*LOCONUV __ __*
**34. Did any doctor say that you have high blood pressure?**<br>(0) No - SKIP TO QUESTION 36<br>(1) Yes - How long: __ __ years __ __ months<br>(99)IGN	*HASREF___*<br>*HASTEMA __ __*<br>*HASTEMM __ __*
**35. Are you taking any medicine recommended or prescribed by your doctor for high blood pressure?**<br>(0) No (1) Yes (8)NSA<br>(9)IGN	*HREMED __*
**36. Did any doctor say that you have diabetes or high blood sugar?**<br>(0) No - SKIP TO QUESTION 38<br>(1) Yes - How long: __ __ years __ __ months<br>(99)IGN	*DIAREF___*<br>*DIATEMA __ __*<br>*DIATEMM __ __*
**37. Are you taking any medicine recommended or prescribed by the doctor for diabetes?**<br>(0) No (1) Yes (8)NSA<br>(9)IGN	*DIAREMED __*
**38. Did any doctor say that you have a lung problem (bronchitis, emphysema, COPD, asthma)?**<br>(0) No - SKIP TO QUESTION 40<br>(1) Yes - How long: __ __ years __ __ months<br>(99)IGN	*PULREF___*<br>*PULTEMA __ __*<br>*PULTEMM __ __*
**39. Are you taking any medicine recommended or prescribed by the doctor for the lung problem (bronchitis, emphysema, COPD, asthma)?**<br>(0) No (1) Yes (8)NSA<br>(9)IGN	*PULREMD__*
**40. In this year (2008) did you get the flu vaccine?**<br>(0) No. Why not?____<br>(1) Yes. Where?______________________________<br>(9)IGN	*VACGRIPE__*<br>*VACNÃOPQ__*<br>*VACONDE__*
**41. <IN THE LAST 10 YEARS> did you get the tetanus vaccine?**<br>(0) No (1) Yes<br>(9)IGN	*VACTET__*

**42. Did any doctor say that you have a heart problem?**<br>(0) No - SKIP TO QUESTION 44<br>(1) Yes - How long: __ __ years __ __ months.<br>(99)IGN	*CORREF___*<br>*CORTEMA __ __*<br>*CORTEMM __ __*
**43. Are you taking any medicine recommended or prescribed by the doctor for the heart problem?**<br>(0) No (1) Yes (8)NSA<br>(9)IGN	*CORREMED __*
**44. Did any doctor say that you had a stroke or stroke?**<br>(0) No<br>(1) Yes - How long: __ __ years __ __ months<br>(99)IGN	*DERREF___*<br>*DERTEMA __ __*<br>*DERTEMM __ __*
**45. Did any doctor say that you have a spinal disease?**<br>(0) No<br>(1) Yes - How long: __ __ years __ __ months<br>(99)IGN	*COLREF___*<br>*COLTEMA __ __*<br>*COLTEMM __ __*
**46. Did any doctor say that you have rheumatism, arthritis or osteoarthritis?**<br>(0) No<br>(1) Yes - How long: __ __ years __ __ months<br>(99)IGN	*RAAREF___*<br>*RAATEMA __ __*<br>*RAATEMM __ __*
**47. Did any doctor say that you have a kidney problem?**<br>(0) No<br>(1) Yes - How long: __ __ years __ __ months<br>(99)IGN	*RIAREF__*<br>*RITEMA__ __*<br>*RITEMM__ __*
**48. Are you undergoing hemodialysis?**<br>(0) No<br>(1) Yes - How long: __ __ years __ __ months<br>(99)IGN	*HEMOD __*<br>*HEMTEMA__ __*<br>*HEMTEMM __ __*
**49. Did any doctor ever tell you that you had cancer?**<br>(0) No - SKIP TO QUESTION 52<br>(1) Yes - How long: __ __ years __ __ months (88)NSA<br>(99)IGN<br>Where in the body:___________________ (88)NSA<br>(99)IGN<br>How long: __ __ years __ __ months (88)NSA<br>(99)IGN<br>Where in the body:___________________ (88)NSA<br>(99)IGN	*CAREF___*<br>*CATEMA1 __ __*<br>*CATEM1 __ __*<br>*CALUG1__ __*<br>*CATEMA2 __ __*<br>*CATEM2 __ __*<br>*CALUG2__ __*
**50. Are you currently undergoing any cancer treatment?**<br>(0) No - SKIP TO QUESTION 52<br>(1) Yes - How long: __ __ years __ __ months (8)NSA (9)IGN	*TCAREF___*<br>*TCATEMA __ __*<br>*TCATEMM __ __*
**51. IF YES, what treatment?**<br>(1) **Chemotherapy**<br>(2) **Radiotherapy**<br>(3) **Other**____ (8)NSA (9)IGN	*TIPOTRATCA __*
**52. Have you ever had to amputate any part of your body in your life?**<br>(0) No<br>(1) Yes - How long: __ __ years __ __ months<br>What part of the body?________________________________ (9)IGN	*AMPREF___*<br>*AMPTEMA __ __*<br>*AMPTEMM __ __*<br>*AMPLUG __ __*
**53. Do you have a problem losing some urine and getting wet accidentally (there is no time to get to the bathroom, or when you are sleeping; or when you cough or sneeze, or force yourself)?**<br>(0) No - SKIP TO QUESTION 57 (1) Yes (9)IGN	*INCURIN __*
**54. <IN THE LAST 30 DAYS> how often did this happen?**<br>(1) **Once or twice a day**<br>(2) **More than twice a day**<br>(3) **Once or twice a week**<br>(4) **More than twice a week**<br>(5) **Once or twice a month**<br>(6) **More than twice a month** (8)NSA (9)IGN	*FREINCURIN __*

**55. Due to your problem of losing a little urine and accidentally getting wet, do you have to wear a diaper (liner, absorbent)?**<br>(0) No - SKIP TO QUESTION 57 (1) Yes (8)NSA<br>(9)IGN	*PROPERUR__*
**56. IF YES, you wear a diaper (liner, absorbent):**<br>**(1) Just to leave**<br>**(2) Only for sleeping**<br>**(3) All the time** (8)NSA<br>(9)IGN	*FRALDA __*
**NOW LET'S TALK ABOUT THE USE OF MEDICINE (MEDICINES)**	
**57. Now let's talk about any medicine you have used <IN THE LAST 7 DAYS>. It can be a remedy for headache, high blood pressure or another remedy that you always use or only occasionally.**<br>**<**IN THE LAST 7 DAYS**>, did you use any medicine?**<br>(0) No - SKIP TO QUESTION 65 (1) Yes<br>(9)IGN	*TOREMED__*
**58. Could you bring the boxes or packages of all the medicines you took <**IN THE LAST 7 DAYS**>?** (88)NSA	

**MEDICATION (NAME)**	**How many days have you been taking it?**	**How many times a day?**	**How many pills do you take at the same time?**	**How many times have you forgot ten to take it?**	**What reason are you taking for?**	**It works 1-Very well 2-Well 3-Not very well**	
							MEDIC1__<br>*TEMPMEDM1__ __*<br>*TEMPMEDD1__ __*<br>*VEZDIAMED1__*<br>*VEZESQMED1__*<br>*MOTIVMED1__*<br>*FUNCIMED1__*
1.__________________ __________________	_ _ **month**<br>_ _ **day**						*MEDIC2__*<br>*TEMPMEDM2__ __*<br>*TEMPMEDD2__ __*<br>*VEZDIAMED2__*<br>*VEZESQMED2__*<br>*MOTIVMED2__*<br>*FUNCIMED2__*
2.__________________ __________________	_ _ **month**<br>_ _ **day**						
3.__________________ __________________	_ _ **month**<br>_ _ **day**						*MEDIC3__*<br>*TEMPMEDM3__ __*<br>*TEMPMEDD3__ __*<br>*VEZDIAMED3__*<br>*VEZESQMED3__*<br>*MOTIVMED3__*<br>*FUNCIMED3__*
4.__________________ __________________	_ _ **month**<br>_ _ **day**						
5.__________________ __________________	_ _ **month**<br>_ _ **day**						*MEDIC4__*<br>*TEMPMEDM4__ __*<br>*TEMPMEDD4__ __*<br>*VEZDIAMED4__*<br>*VEZESQMED4__*<br>*MOTIVMED4__*<br>*FUNCIMED4__*
6.__________________ __________________	_ _ **month**<br>_ _ **day**						
7.__________________ __________________	_ _ **month**<br>_ _ **day**						*MEDIC5__*<br>*TEMPMEDM5__ __*<br>*TEMPMEDD5__ __*<br>*VEZDIAMED5__*<br>*VEZESQMED5__*<br>*MOTIVMED5__*<br>*FUNCIMED5__*
8.__________________ __________________	_ _ **month**<br>_ _ **day**						
9.__________________ __________________	_ _ **month**<br>_ _ **day**						*MEDIC6__*<br>*TEMPMEDM6__ __*<br>*TEMPMEDD6__ __*<br>*VEZDIAMED6__*<br>*VEZESQMED6__*<br>*MOTIVMED6__*<br>*FUNCIMED6__*
10.__________________ __________________	_ _ **month**<br>_ _ **day**						
**Total number of medications:__ __**							*NUTOMED__ __*

**59. Do any of the remedies bother you in any way?**<br>(0) No - SKIP TO 61 (1) Yes (8)NSA (9)IGN	*REMEDINC__*

60. Can you tell me which medicine(s) (how much and how much do you bother)?

Medicine Name	How much it Does it bother you?			Where it bothers you?	
	A lot	A little	Not a lot		
1.____________					*REMED1__*<br>*INCREMED1__*<br>*QINCOMED1__*
2.____________					*REMED2__*<br>*INCREMED2__*<br>*QINCOMED2__*
3.____________					*REMED3__*<br>*INCREMED3__*<br>*QINCOMED3__*
4.____________					*REMED4__*<br>*INCREMED4__*<br>*QINCOMED4__*
5.____________					*REMED5__*<br>*INCREMED5__*<br>*QINCOMED5__*

61. Now I am going to read some problems that people have when taking their medicine and I would like you to tell me if it is "very difficult", "a little difficult" or if "it is not difficult" to do each one of the tasks.

Task	Very difficul	A littl difficu t	Not difficult	Comments (which medicine)	
1. Remove the medicine from the packaging					*RETREM__*
2. Read the medicine package					*LERREM__*
3. Remember to take all medications					*LEMBREM__*
4. Being able to replace medicines in time					*CONSREM__*
5. Take too many medications at the same time					*TOMUREM__*

**62. How do you get these remedies <u>most of the time</u>?**<br>(1) Primary Health Care Unit. Which one?______________________<br>(2) Health Office<br>(3) You have to buy - APPLY QUESTION 63, IF YOU DON'T JUMP FOR 64<br>(4) Got part of the medication and another part has to buy - APPLY QUESTION 63<br>(5) Other:________________________________ (8)NSA (9)IGN	*REMAVZ__*<br>*REMAVZPS__*
**63. If you had to buy, how much have you spent on medication since <LAST 30 DAYS>?**<br>**R$: __ __ __ __, __ __**<br>(8888,88)NSA (9999,99)IGN	*REMCOMP*<br>__ __ __ __,__ __
**64. Did you have any medication that you had to take since <LAST 30 DAYS> and you didn't get it?**<br>(0) No (1) Yes (8)NSA (9)IGN	*RETONC__*
**65. Have you ever fallen from <1 YEAR AGO> until now?**<br>(0) No - SKIP TO QUESTION 67 (1) Yes (9)IGN	*QUEULTA__*
**66. IF YES - How many times??** __ __ times<br>(88)NSA (99)IGN	*QUEVZA__ __*
**67. Since <1 YEAR AGO> have you broken or broken any bones?**<br>(0) No - SKIP TO QUESTION 69 (1) Yes<br>(9)IGN	*FRAT__*

**68. IF YES - How many times**? __ __ vezes<br>(88)NSA (99)IGN	*FRATVZA__ __*
**69. Since <4 YEARS AGO>, did you have to be hospitalized (discharged) in any hospital?**<br>(0) No - SKIP TO QUESTION 71 (1) Yes (9)IGN	*INT4ANO__*
**70. IF YES, how many times? __ __**(number of times) (8)NSA (9)IGN	*INT4AVZ__ __*
**71. Since <1 YEAR AGO>, did you have to be hospitalized (discharged) in any hospital?**<br>(0) No - SKIP TO QUESTION 74 (1) Yes (9)IGN	*INTEULTA__*
**72. IF YES, what is the reason for the last hospitalization**?<br>____ (8)NSA (9)IGN	*INTMOT__ __*
**73. IF YES, how many times? __ __**(number of times) (8)NSA (9)IGN	*INTULTMVZ__ __*
**74. Since <1 YEAR AGO>, did you need to spend the night in a hospital under observation (as a patient)?**<br>(0) No - SKIP TO QUESTION 76 (1) Yes (9)IGN	*NOIHOSUA__*
**75. IF YES, how many times? __ __**(number of times) (8)NSA (9)IGN	*NOIHOSVZ__ __*
**76. Have you ever consulted your eyes with an eye specialist, doctor or technician in your life? (excluding exams for taking or renewing a driver's license)**<br>(0) No - SKIP TO QUESTION 78 (1) Yes (9)IGN	*CONSOLHOS__*
**77. When was the last time you consulted for the eyes?**<br>(0) less than 1 year ago<br>(1) Between 1 and 5 years ago<br>(2) More than 5 years ago<br>(9) Doesn't remember how long (8)NSA (9)IGN	*VICONSOLHT__*
**78. Do you wear glasses or contact lenses?**<br>(0) No - SKIP TO QUESTION 80<br>(1) Yes - How long ago? __ __years (99)IGN	*VISOCLEN__*<br>*VITEOCLEN__ __*
**79. Was this glasses or contact lens prescribed by a health professional?**<br>(0) No (1) Yes (8)NSA (9)IGN	*VIOCLENMED__*
**80. Do you consider your vision? (with or without glasses or lens)**<br>*SHOW THE FACES CARD!*<br>**(1) great (2) good (3) fair (4) bad (5) terrible** (9)IGN	*VISAO__*
**81. Does your vision hinder you to do the things you need or want to do?**<br>(0) No (1) Yes (9)IGN	*VIATRP__*
**82. How do you consider your hearing? (hear well? listen well?) (with or without the help of devices)**<br>*SHOW THE FACES CARD!*<br>**(1) great (2) good (3) fair (4) bad (5) terrible** (9)IGN	*AUDI__*
**83. Do you use hearing aids?**<br>(0) No<br>(1) Yes, how long ago? __ __ (months) (9)IGN	*AUDIAP__*<br>*AUDIAPTE__ __*
**84. Does your hearing disturb you for the activities you need or want to do?**<br>(0) No<br>(1) Yes (9)IGN	*AUDIATRP__*
**85. How do you consider the situation of your mouth?**<br>*SHOW THE FACES CARD!*<br>(**(1) great (2) good (3) fair (4) bad (5) terrible** (9)IGN	*ODCA__*
**86. Have you ever consulted a dentist in your life?**<br>(0) No - SKIP TO QUESTION 90<br>(1) Yes (9)IGN	*ODCONS__*
**87. How long ago was the last appointment with the dentist?**<br>(1) Less than 1 year ago<br>(2) Between 1 and 5 years ago<br>(3) More than 5 years ago<br>(4) Doesn't remember how long (8)NSA (9)IGN	*ODCAULT__*

**88. What is the main reason (s) for the last time you consulted with the dentist?**<br>**(0) Routine / maintenance**<br>**(1) I was in pain**<br>**(2) Had bleeding or inflamed gums**<br>**(3) Had caries / restoration / filling**<br>**(4) Had any sores, lumps or stains in the mouth**<br>**(5) My face was swollen**<br>**(6) Needed to undergo root canal treatment**<br>**(7) I needed to pull out some teeth**<br>**(8) I had to make a new denture**<br>**(9) Others**_________________ (88)NSA (99)IGN	*ODMOTC1*__ __<br>*ODMOTC2*__ __<br>*ODMOTC3*__ __
**89. Where did you see the dentist?**<br>**(0) At the Primary Health Care Center - Which one?**______________________<br>**(1) Private dentist**<br>**(2) Private Ambulatory**<br>**(3) Contracted dentist**<br>**(9) Other**_____________________ (88)NSA (99)IGN	*ODLOC*__ __<br>*PSQUAL*__ __
**90. Do you have a problem or difficulty chewing food?**<br>(0) No (1) Yes (9)IGN	*ODIFMAS*__
**91. <IN THE LAST 30 DAYS> did you need to stay in bed (were you bedridden)?**<br>(0) No - SKIP TO QUESTION 93<br>(1) Yes (9)IGN	*ACAM30D*__
**92. How long were you in bed?** __ __ (months)__ __ (days)<br>(88)NSA (99)IGN	*TEMACM*__ __<br>*TEMACD*__ __
**93. Have you ever been visited by a Community Health Agent (CHA) at your home?**<br>(0) No □ SKIP TO QUESTION 96 (1) Yes (9)IGN	*VISACS*__
**94. IF YES, you received a visit from the CHA at your home at:**<br>*<IN THE LAST 30 DAYS>* (0)No<br>(1)Yes, __ __ times (8)NSA (9)IGN<br><br>***<**FROM 3 MONTHS AGO**>*** (0)No<br>(1) Yes, __ __ times (8)NSA (9)IGN	*VACS30D*__<br>*VACS30DV*__ __<br><br>*VACS3M*__<br>*VACS3M*__ __
**95. Which of the activities will I read, did the Community Health Worker (Agente Comunitário de Saúde – ACS) do at your home?**	
**Filled out a form for your registration** (0)No (1)Yes (8)NSA (9)IGN	*CAD*__
**Asked about his health situation** (0)No (1)Yes (8)NSA (9)IGN	*SITSAU* __
**Asked about medication use** (0)No (1)Yes (8)NSA (9)IGN	*USOMED*__
**Delivered medications or dressing material** (0)No (1)Yes (8)NSA (9)IGN	*ENTMED*__
**Oriented about vaccines** (0)No (1)Yes (8)NSA (9)IGN	*VACONS*__
**Guided on the importance of cleaning the mouth and prosthesis** (0)No (1)Yes (8)NSA (9)IGN	*ORITBOCA*__
**Provided guidance on health care** (0)No (1)Yes (8)NSA (9)IGN	*ORITSAU*__
**Scheduled appointment** (0)No (1)Yes (8)NSA (9)IGN	*AGENDCONS*__
**NOW LET'S TALK ABOUT HEALTH CARE AT HOME**	
**96. Since <THREE MONTHS AGO> you have received at home any of the following services:**	
**Medical consultation?** (0)No (1)Yes (9)IGN	*RECONMED*__
**Social assistance?** (0)No (1)Yes (9)IGN	*RECASSISOC*__
**Physiotherapy?** (0)No (1)Yes (9)IGN	*RECFIS*__
**Dentist care?** (0)No (1)Yes (9)IGN	*RECATDENT*__
**Nursing care?** (0)No (1)Yes (9)IGN	*RECATENF*__
**Pressure check?** (0)No (1)Yes (9)IGN	*RECATA*__
**Band Aid?** (0)No (1)Yes (9)IGN	*RECCUR*__
**Injection?** (0)No (1)Yes (9)IGN	*RECINJ*__
**Flu vaccine application?** (0)No (1)Yes (9)IGN	*RECVAC*__
**Nebulization?** (0)No (1)Yes (9)IGN	*RECNEB*__
**Bladder catheterization?** (0)No (1)Yes (9)IGN	*RECSONVES*__
**Was material collected for exams (eg blood)?** (0)No (1)Yes (9)IGN	*RECOLEX*__
**Other**__________________________________	*RECOU*__
**IF YES IN ANY OF THE ABOVE QUESTIONS, APPLY QUESTIONS 97, 98 and 99;**<br>**kyyyr**	
**97. Why did you need health care at home?**	

**Was bedridden** (0)No (1)Yes (8)NSA (9)IGN<br>**I had difficulty walking** (0)No (1)Yes (8)NSA (9)IGN<br>**Her health situation had worsened** (0)No (1)Yes (8)NSA (9)IGN<br>**Needed follow-up after discharge from hospital** (0)No (1)Yes (8)NSA (9)IGN<br>**There was no one to take you to the health center** (0)No (1)Yes (8)NSA (9)IGN<br>**Other:** ________________________	*MOTADAC__*<br>*MOTADDL__*<br>*MOTADSP__*<br>*MOTADSP__*<br>*MOTADNA__*<br>*MOTIVOU__*
**98. How many times have you received health care at home since <3 MONTHS AGO>? __ __**<br>**How many of these were <IN THE LAST MONTH>? __ __**<br>**How many of these were <IN THE LAST WEEK>? __ __** (88) NSA (99)IGN	*PREAD3M__*<br>*PREADULM__ __*<br>*PREADULS__ __*
**99. Did you receive care from any of the following:**<br>**Health professional from Primary Health Care Center** (0)No (1)Yes (8)NSA (9)IGN<br>**Private professional** (0)No (1)Yes (8)NSA (9)IGN<br>**Health insurance professional** (0)No (1)Yes (8)NSA (9)IGN<br>**From your family member** (0)No (1)Yes (8)NSA (9)IGN<br>**From a neighbor or friend** (0)No (1)Yes (8)NSA (9)IGN<br>**Other:**________________________	*REPROPSAU__*<br>*REPROFPAR__*<br>*REPPROCON__*<br>*REFAMILIAR__*<br>*REVIZAMIGO__*<br>*REAOUT__*
**100. Have you been asked for home care since <3 MONTHS AGO>?**<br>(0) No (1) Yes - SKIP TO QUESTION 102 (9) IGN	*SOLAD__*
**101. For what reason did you not request care at home?**<br>**The service does not provide care at home** (0)No (1)Yes (8)NSA (9)IGN<br>**No professional to attend at home** (0)No (1)Yes (8)NSA (9)IGN<br>**The service does not have a telephone or does not work** (0)No (1)Yes (8)NSA (9)IGN<br>**There was no way to make an appointment or request an appointment** (0)No (1)Yes (8)NSA (9)IGN<br>**Was afraid to request and not be attended** (0)No (1)Yes (8)NSA (9)IGN<br>**Why I feel better** (0)No (1)Yes (8)NSA (9)IGN<br>**Other:** ________________________	*MONSADNF__*<br>*MONSADNTP__*<br>*MONSADNTT__*<br>*MONSADNTC__*<br>*MONSADTM__*<br>*MONSADMEL__*<br>*MONSADOUT__*
**102. IF YES: Where did you request home care?**<br>**(0) Primary Health Care Center. Which one?**________________________<br>**(1) At the Municipal Health Secretariat**<br>**(2) Emergency Movel Service**<br>**(3) In the health insurance plan**<br>**(4) Outpatient or private service**<br>**(5) Other:** ________________________ (8)NSA (9)IGN	*ONSOLAD__*<br>*PSQUAL __ __*<br>*SADOUT__*
**103. IF YES: Who made the request for home care?**<br>**You** (0)No (1)Yes (8)NSA (9)IGN<br>**Any of your family members** (0)No (1)Yes (8)NSA (9)IGN<br>**Any neighbor or friend** (0)No (1)Yes (8)NSA (9)IGN<br>**The Community Health Worker - ACS** (0)No (1)Yes (8)NSA (9)IGN<br>**Other:**________________________	*ADSOLS__*<br>*ADSOLF__*<br>*ADSOLVA__*<br>*ADSOLACS__*<br>*ADSOLOU__*
**104. IF YES: How did you apply?**<br>**Over the phone** (0)No (1)Yes (8)NSA (9)IGN<br>**Some family member or neighbor came to the service** (0)No (1)Yes (8)NSA (9)IGN<br>**Asked the ACS** (0)No (1)Yes (8)NSA (9)IGN<br>**Other:**________________________	*FSOADSE__*<br>*FSOLSER__*<br>*FSOLACS__*<br>*FSOLOU__*
**105. Did you receive the requested service?**<br>(0) No - SKIP TO QUESTION 108 (1) Yes (8)NSA (9) IGN	*RECEBEU__*
**106. How many days have passed between the request and the professionals coming to your home?**<br>__ __ (days) (88)NSA (99)IGN	*QTSOLAD__ __*

**107. What is your opinion about the waiting time to be attended at home this last time?**<br>*SHOW THE FACES CARD!*<br>**1) Poor (2) Bad (3) Regular (4) Good (5) Great** (8)NSA (9)IGN	*OPINTEAD__*
**108. IF NO: For what reason was it not attended to?**<br>**Could not get a record in the service** (0)No (1)Yes (8)NSA (9)IGN<br>**The service does not answer at home** (0)No (1)Yes (8)NSA (9)IGN<br>**There was no response from the service** (0)No (1)Yes (8)NSA (9)IGN<br>**The service had no professional to attend** (0)No (1)Yes (8)NSA (9)IGN<br>**The service was closed** (0)No (1)Yes (8)NSA (9)IGN<br>**Needed to pay and had no money** (0)No (1)Yes (8)NSA (9)IGN<br>**The phone was always busy** (0)No (1)Yes (8)NSA (9)IGN<br>**( ) Other:**________________________	 *MONRADF__*<br>*MONRADNF__*<br>*MONRADNR__*<br>*MONRADNTP__*<br>*MONRADPF__*<br>*MONRADNPP__*<br>*MONRADTO__*<br>*MONRADOU__*
**109. IF NOT RECEIVED: What happened to your health situation?**<br>**(1) Still in the same situation (2) Improved (3) Worsened**<br>**( ) Other:** ____ (8)NSA (9)IGN	*PRENROQAC__*
**FOR THOSE WHO HAVE NOT RECEIVED HOME CARE CLOSE HERE AND CONTINUE WITH QUESTION 126**	
**110. How many times have you been attended to at home in the last three months by staff from...**<br><br>**Primary Health Care Center in your neighborhood:** __ __<br>(88)NSA (99)IGN<br>**How many times in the last month?** __ __<br>(88)NSA (99)IGN<br>**How many in the last week?** __ __<br>(88)NSA (99)IGN	 <br><br>*ADQVPSB__ __*<br>*ADQVPSBUM__ __*<br>*ADQVPSBUS__ __*
**Primary Health Care Center in another neighborhood:**<br>**Whichl**?______________________________ (88)NSA (99)IGN<br>**How many times in the last month?** __ __ (88)NSA (99)IGN<br>**How many times in the last week?** __ __ (88)NSA (99)IGN	 *ADQVPSOB__ __*<br>*ADQVPSOBUM__ __*<br>*ADQVPSOBUS__ __*
**SAMU:** __ __ times<br>**How many times in the last month?** __ __ (88)NSA (99)IGN<br>**How many times in the last week?** __ __ (88)NSA (99)IGN	*ADQVSAMU __ __*<br>*ADQVSAMUUM__ __*<br>*ADQVSAMUUS__ __*
**Other:**___________________________ (88)NSA (99)IGN<br>**How many times in the last month?** __ __ (88)NSA (99)IGN<br>**How many times in the last week?** __ __ (88)NSA (99)IGN	*ADQVOUT__ __*<br>*ADQVOUTUM__ __*<br>*ADQVOUTUS__ __*
**NOW LET'S TALK ABOUT <u>THE LAST TIME</u> HE RECEIVED HEALTH CARE AT HOME**	
**111. Which professionals attended you at home this last time?**<br>**Nurse** (0)No (1)Yes (8)NSA (9)IGN<br>**Nursing Assistant / Technician** (0)No (1)Yes (8)NSA (9)IGN<br>**Physician** (0)No (1)Yes (8)NSA (9)IGN<br>**Dentist** (0)No (1)Yes (8)NSA (9)IGN<br>**Physiotherapist** (0)No (1)Yes (8)NSA (9)IGN<br>**Nutritionist** (0)No (1)Yes (8)NSA (9)IGN<br>**Psychologist** (0)No (1)Yes (8)NSA (9)IGN<br>**Physical Educator** (0)No (1)Yes (8)NSA (9)IGN<br>**Speech therapist** (0)No (1)Yes (8)NSA (9)IGN<br>**Social Worker** (0)No (1)Yes (8)NSA (9)IGN<br>**Student** (0)No (1)Yes (8)NSA (9)IGN<br>**Outro:**___________________________	 *ADUVENF__*<br>*ADUVTENF__*<br>*ADUVMED__*<br>*ADUVOD__*<br>*ADUVFIS__*<br>*ADUVNUT__*<br>*ADUVPSI__*<br>*ADUVEDFI__*<br>*ADUVFON__*<br>*ADUVASS__*<br>*ADUVEST__*<br>*ADUVOUT__*
**112. What was done with you during home care this last time?**<br>**Medical consultation** (0)No (1)Yes (8)NSA (9)IGN<br>**Did physiotherapy** (0)No (1)Yes (8)NSA (9)IGN<br>**Nursing consultation** (0)No (1)Yes (8)NSA (9)IGN<br>**Dressing** (0)No (1)Yes (8)NSA (9)IGN<br>**Nebulization** (0)No (1)Yes (8)NSA (9)IGN<br>**Injected** (0)No (1)Yes (8)NSA (9)IGN<br>**Measured blood pressure** (0)No (1)Yes (8)NSA (9)IGN<br>**Measured temperature** (0)No (1)Yes (8)NSA (9)IGN<br>**They changed the ostomy "bag"** (0)No (1)Yes (8)NSA (9)IGN<br>**Placed / exchanged urethral tube** (0)No (1)Yes (8)NSA (9)IGN<br>**Nasogastric / naso-enteral tubes were placed / exchanged** (0)No (1)Yes (8)NSA (9)IGN	 *ADCMED__*<br>*ADFISIO__*<br>*ADCENF__*<br>*ADCUR__*<br>*ADNEB__*<br>*ADMEDIN__*<br>*ADMPA__*<br>*ADMTEM__*<br>*ADTBOL__*<br>*ADTSON__*<br>*ADSNG __*

**Blood sugar levels** (0)No (1)Yes (8)NSA (9)IGN	*ADDAS__*
**Applied flu vaccine** (0)No (1)Yes (8)NSA (9)IGN	*ADVACG__*
**Applied tetanus vaccine** (0)No (1)Yes (8)NSA (9)IGN	*ADVACT__*
**Teeth cleaning** (0)No (1)Yes (8)NSA (9)IGN	*ADLIMPD__*
**Tooth filling** (0)No (1)Yes (8)NSA (9)IGN	*ADOBD__*
**Tooth extraction (plucking)** (0)No (1)Yes (8)NSA (9)IGN	*ADEXD__*
**Adjustment or making of prosthesis, pivot, denture** (0)No (1)Yes (8)NSA (9)IGN	*ADPPD__*
**Other** ______________________	*ADOUT__*
**113. Did you remain in follow-up after this service?** (0) No - SKIP TO QUESTION 115 (1) Yes (8)NSA (9)IGN	*PEACAD__*
**114. If YES, your follow-up, in most cases was:**	
**Daily** (0)No (1)Yes (8)NSA (9)IGN	*READDI__*
**Once a week** (0)No (1)Yes (8)NSA (9)IGN	*READUVS__*
**2 or more times a week** (0)No (1)Yes (8)NSA (9)IGN	*READDVS__*
**1 time every fifteen days** (0)No (1)Yes (8)NSA (9)IGN	*READUQD__*
**Once a month** (0)No (1)Yes (8)NSA (9)IGN	*READUVM__*
Other:___________________________	*READOUT__*
**115. After the consultation do you was**	
**Referred to hospital** (0)No (1)Yes (8)NSA (9)IGN	*ENCHOS__*
**Referred to specialist** (0)No (1)Yes (8)NSA (9)IGN	*ENCESP__*
**Exam requested** (0)No (1)Yes (8)NSA (9)IGN	*SOLEX__*
**New drug prescription** (0)No (1)Yes (8)NSA (9)IGN	*PRESNM__*
**Health Care Oriented** (0)No (1)Yes (8)NSA (9)IGN	*DISPMAT__*
**Left material or equipment** (0)No (1)Yes (8)NSA (9)IGN	*DEIXMAT__*
**If YES, apply to 116, if NOT to jump to 117**	
**116. What materials or equipment did the Health Center staff leave at your home for your care?** (0) gauze (1) syringe (2) medications (3) cotton (4) adhesive tape (5) gloves (6) bladder catheter ( ) Other:______________________________ (8) NSA (9) IGN	*MATEQD1__* *MATEQD2__* *MATEQD3__* *MATEQD4__*
**117. Did you receive any explanation about the reason for your attendance at home?** (0) No (1)Yes (8) NSA (9) IGN	*RECEXPMOT__*
**118. Did you spend any money on the last appointment you received at home?** (0) No - SKIP TO QUESTION 120 (1)Yes (8) NSA (9) IGN	*GADICA__*
**119. If YES: How much did you spend?** R$__ __ __ __, __ __ (8888,88) NSA (9999,99) IGN	*GADIQT_ _ _ _, _ _*
**120. Did you receive a prescription for this last home care?** (0) No - SKIP TO QUESTION 124 (1) Yes (8) NSA (9) IGN	*RECRECUAC__*
**121. Did you get the medicines through SUS?** (0) Não (1)Sim (8) NSA (9) IGN	*CONSREMSUS__*
**122. IF NO: Did you buy any medicine?** (0) No - SKIP TO QUESTION 124 (1) Yes (8) NSA (9) IGN	*COMPREM__*
**123. IF YOU BUY: How much did you spend?** R$:__ __ __ __, __ __ (8888,88) NSA (9999,99) IGN	*QUANTOGAS_ _ _ _, _ _*

**124. What is your opinion about the health care you received at home this last timez?**<br>*SHOW THE FACES CARD!*<br>**(1) Poor (2) Bad (3) Regular (4) Good (5) Great** (8)NSA (9)IGN	*OPIADUV*__
**125. After receiving health care at home, you consider that your problem:**<br>**(0) Worsened**<br>**(1) Same**<br>**(2) Improved a little**<br>**(3) Much improved**<br>**(4) Cured / resolved** (8)NSA (9)IGN	*AADPROB*__
**NEXT I WILL ASK YOU ABOUT SOME ACTIVITIES OF YOUR DAY AND I WOULD LIKE YOU TO RESPOND TO ME ACCORDING TO THE ALTERNATIVES I WILL GIVE YOU**	
**126. When are you going to take your shower:**<br>**(2) Does not receive help (enters and leaves the bathroom alone)**<br>**(1) Receives help with bathing for only one part of the body (back or legs, for example)**<br>**(0) Receives help with bathing in more than one part of the body**	*IBANHO* __
**127. When will you get dressed:**<br>**(2) Does not receive help**<br>**(1) Get the clothes and get dressed without help (except to tie your shoes)**<br>**(0) Receives help to pick up clothes or to dress (or remains partially or totally undressed)**	*IVESTIR* __
**128. When you need to use the bathroom for your needs:**<br>**((2) Does not receive help**<br>**(1) Receives help to go to the bathroom**<br>**(0) Do not go to the bathroom to urinate or evacuate**	*ITOALET* __
**129. To move from the bed to a chair, you:**<br>**(2) Does not receive help**<br>**(1) Receives help**<br>**(0) Don't get out of bed**	*ICADEIR* __
**130. Do you have control to pee or poop:**<br>**(2) Has control over urination and evacuation functions**<br>**(1) There are occasional 'accidents'**<br>**(0) Can not control the pee or poop and wear a diaper or tube**	*ICAMIN* __
**131. To feed (to eat):**<br>**(2) Feeds without assistance**<br>**(1) Feeds without help, except to cut meat or butter bread**<br>**(0) Receives help to feed or is fed by tube**	*IALIMEN* __
**132. To use the telephone?**<br>**(2) Has no difficulty**<br>**(1) Can do with difficulty**<br>**(0) Cannot use alone**	*ITELEF* __
**133. To go to distant places, using bus or taxi, you:**<br>**(2) Does not receive help**<br>**(1) Receives partial aid**<br>**(0) Can't go alone**	*ISAIR* __
**134. To make your purchases, you:**<br>**(2) Does not receive help**<br>**(1) Receives partial aid**<br>**(0) Can't do it alone**	*ICOMPR* __
**135. To prepare your own meals, you:**<br>**(2) Does not receive help**<br>**(1) Receives partial aid**<br>**(0) Cannot prepare alone**	*ICOMIDA* __
**136. To clean your house**<br>**(2) Does not receive help**<br>**(1) Receives partial aid**<br>**(0) Can't fix it alone**	*ILIMPEZ* __
**137. To handle small objects, such as a key, or to make minor repairs or housework**<br>**(2) Does not receive help**<br>**(1) Receives partial aid**	*IOBJPEQ* __

(0) Can't do it alone	
**138. To take your medication at the right dose and at the right time**<br>((2) Does not receive help<br>(1) Receives partial aid<br>(0) Can't take it alone	*IREMED* __
**139. To take care of your money**<br>(2) Does not receive help<br>(1) Receives partial aid<br>(0) Can't manage alone	*IDINHE* __
**140. To walk the distance of a block**<br>(2) Does not receive help<br>(1) Receives partial aid<br>(0) Can't walk alone	*ICAQUA* __
**141. To climb a flight of stairs**<br>(2) Does not receive help<br>(1) Receives partial aid<br>(0) Cannot climb alone	*ILANCE* __
**IF THE INTERVIEWER ANSWERED THAT THEY NEED PARTIAL HELP OR GREAT HELP IN THE ABOVE QUESTIONS, APPLY QUESTION 142. IF NO, JUMP TO QUESTION 145**	
**142. Who do you get help with in most tasks that need it?**<br>**(1) Partner; spouse -** If the elderly DO NOT HAVE A COMPANION, DO NOT READ THIS OPTION<br>**(2) Child –** *IF the elderly do not HAVE CHILDREN, DO NOT READ THIS*<br>**(3) Neighbor**<br>**(4) Friends**<br>**(5) Paid companion - APPLY QUESTION 143**<br>**(6) Unpaid companion - SKIP TO QUESTION 144**<br>**(7) Other**________________________________ (8)NSA (9)IGN	*RECAJU* __
**143. If you pay, how much do you pay per month?**<br>R$__ __ __ __, __ __(reais) (888888)NSA (999999)IGN	PAGME<br>_ _ _ _ , _ _
**144. How long (in hours) do you receive help during the day?**<br>__ __ (hours)__ __(min) (00) less than 1 hour (88)NSA (99)IGN	TEAJHD __ __<br>TEAJMD__ __
**SOCIAL RELATIONSHIP AND SUPPORT NETWORK**	
**145. During a normal week, in the <LAST 30 DAYS>, did you leave the house (outside the building)?**<br>(0) No day out<br>(1) Left every day<br>(2) Left once a week<br>(3) Left 2 to 4 times a week (9)IGN	*SAIUCASA* __
**146. <IN THE LAST 15 DAYS> did you visit your family?**<br>(0) No - SKIP TO QUESTION 148<br>(1) Yes<br>(2) No family - JUMP FOR 150, DO NOT APPLY 156 AND 157 (9)IGN	*VISFAM2S* __
**147. IF YES, how many times?**<br>(1) Once or twice<br>(2) Three to six times<br>(3) More than six times (8)NSA (9)IGN	*VISFAMFR* __
**148. <IN THE LAST 15 DAYS>, did your family visit you?**<br>(0) No - SKIP TO QUESTION 150<br>(1) Yes (8)NSA (9)IGN	*FAMVIS2S* __
**149. IF YES, how many times?**<br>(1) Once or twice<br>(2) Three to six times<br>(3) More than six times (8)NSA (9)IGN	*FAMVISFR* __
**150. <IN THE LAST 15 DAYS> did you visit your friends?**<br>(0) No - SKIP TO QUESTION 152 (1) Yes (9)IGN	*VISAMI2S* __
**151. IF YES, how many times?**<br>(1) Once or twice<br>(2) Three to six times<br>(3) More than six times (8)NSA (9)IGN	*VISAMFR* __
**152. <IN THE LAST 15 DAYS>, did your friends visit you?**<br>No - SKIP TO QUESTION 154 (1) Yes (9)IGN	*AMVIS2S* __
**153. IF YES, how many times?**<br>(1) Once or twice<br>(2) Three to six times<br>(3) More than six times (8)NSA (9)IGN	*AMVISFR* __

**154. <IN THE LAST 15 DAYS>, did you have contact by phone or letter with your relatives or friends?**<br>No - SKIP TO QUESTION 156 (1) Yes (9)IGN	*TELAM2S* __
**155. IF YES, how many times?**<br>((1) Once or twice<br>(2) Three to six times<br>(3) More than six times (8)NSA (9)IGN	*TELAMFR* __
**156. What kind of help or assistance does your family offer to you? (family members who live / or who do not live with the interviewee)**<br>**Cash** (0)No (1)Yes (8)NSA (9)IGN<br>**Home** (0)No (1)Yes (8)NSA (9)IGN<br>**Company / personal care** (0)No (1)Yes (8)NSA (9)IGN<br>Other:___________________________	*TIAJFAMOFD* __<br>*TIAJFAMOFM* __<br>*TIAJFAMOFC* __<br>*TIAJFAMOFO* __
**157. What kind of help or assistance do you offer to your family? (family members who live / or who do not live with the interviewee).**<br>**Cash** (0)No (1)Yes (8)NSA (9)IGN<br>**Home** (0)No (1)Yes (8)NSA (9)IGN<br>**Company / personal care** (0)No (1)Yes (8)NSA (9)IGN<br>Other:___________________________	*TIAJOFFAMD* __<br>*TIAJOFFAMM* __<br>*TIAJOFFAMC* __<br>*TIAJOFFAMO* __
**158. Are you satisfied with the relationship you have with your friends?**<br>(8) The interviewee says he / she does not have friends<br>(0) Not satisfie (1) Yes (9)IGN	*SATISRELAM* __
**159. You are satisfied with the relationship you have with your neighbors**<br>(8) Interviewee says he has no relationship with neighbors<br>(0) Not satisfied (1) Yes (9)IGN	*SATISRELVIZ* __
**160. Do you have any pets in your home?**<br>(0) No- SKIP TO QUESTION 162 (1) Yes (9)IGN	*ANIESTI*__
**161. IF YES, WHICH?**<br>**Cat** (0)No (1)Yes (8)NSA (9)IGN<br>**Dog** (0)No (1)Yes (8)NSA (9)IGN<br>**Bird** (0)No (1)Yes (8)NSA (9)IGN<br>**Horse** (0)No (1)Yes (8)NSA (9)IGN<br>**Other:**____________________________________	*ANIESTI1* __<br>*ANIESTI2* __<br>*ANIESTI3* __<br>*ANIESTI4* __<br>*OUTANIESTI* __
**162. <IN THE PAST WEEK> did you receive a visit from any of these people?**<br>**Neighbors / friends** (0)No (1)Yes (9)IGN<br>**Brother)** (0)No (1)Yes (9)IGN<br>**Child** (0)No (1)Yes (9)IGN<br>**Other family members(nephews, grandchildren)** (0)No (1)Yes (9)IGN<br>**Other:**__________________________	*RECVISVIZAM* __<br>*RECVISIR* __<br>*RECVISF* __<br>*RECVISOURFA* __<br>*RECVISOUT* __
**163. <IN THE LAST 15 DAYS> did you watch television?**<br>(0) No- SKIP TO QUESTION 166 (1) Yes<br>(9)IGN	*ASSISTV*__
**164. When you watch television, what does he like to watch?**<br>**Movie** (0)No (1)Yes (8)NSA (9)IGN<br>**Novel** (0)No (1)Yes (8)NSA (9)IGN<br>**News** (0)No (1)Yes (8)NSA (9)IGN<br>**Games** (0)No (1)Yes (8)NSA (9)IGN<br>**Others:**__________________________________	*ASSISTV1*__<br>*ASSISTV2*__<br>*ASSISTV3*__<br>*ASSISTV4*__<br>*ASSISTVO*__
**165. How many hours a day, more or less, do you usually watch television?**<br>__ __ hours __ __min (88) NSA (99)IGN	*HORTVDIA*__ __<br>*MINTVDIA*__ __
**166. <IN THE LAST 30 DAYS>, did you do any of these activities?**<br>**Church** (0)No (1)Yes (9)IGN<br>**Participated in a party in the community** (0)No (1)Yes (9)IGN<br>**Participated in family party** (0)No (1)Yes (9)IGN<br>**Participated in a workshop or group** (0)No (1)Yes (9)IGN<br>**Attended a ball** (0)No (1)Yes (9)IGN<br>**Traveled to another city** (0)No (1)Yes (9)IGN<br>**Traveled on tour** (0)No (1)Yes (9)IGN<br>**Went to a funeral or burial** (0)No (1)Yes (9)IGN	*MISSA* __<br>*FESCOM* __<br>*FESFA* __<br>*OFIMI* __<br>*BAILE* __<br>*VIAJ* __<br>*EXC* __<br>*VELENT* __
**NOW I WILL ASK SOME QUESTIONS ABOUT PHYSICAL ACTIVITY**	

**167. Since <DAY OF THE PAST WEEK> how many days have you walked for more than 10 consecutive minutes? Think of walking at work, at home, as a form of transportation to get from one place to another, for leisure, for pleasure or as a form of exercise.**<br>____ days (0) none ▯ SKIP TO QUESTION 169 (9) IGN	*CAMDIA* __
**168. On the days you walked, how long, in total, did you walk each day?**<br>__ __ __ minutes per day (888)NSA<br>(999)IGN	*MINCA*__ __ __
**NOW WE'LL TALK ABOUT OTHER PHYSICAL ACTIVITIES OUTSIDE THE WALK**	
**169. Since <DAY OF THE PAST WEEK> how many days did you do STRONG activities, which made you sweat a lot or increase your breathing and your heart rate a lot, for more than 10 minutes in a row? For example: running, exercising, cycling fast, doing heavy household chores at home, in the yard or garden, transporting heavy objects, playing competitive football, ...**<br>_____ days / week (0) none ▯ SKIP TO QUESTION 171 (9)IGN	*FORDIA*__
**170. On the days when you did strong activities, how long, in total, did you do strong activities per day?**<br>__ __ __ minutes per day (888)NSA (999)IGN	*MINFOR*__ __ __
**171. Since <DAY OF THE PAST WEEK> how many days did you do AVERAGE activities, which made you sweat a little or increase your breathing and your heart rate a little, for more than 10 minutes in a row? For example: cycling at medium pace, swimming, dancing, playing sports just for fun, doing light housework, at home or on the patio, like sweeping, vacuuming, etc.**<br>____ days (0) none ▯ SKIP TO ISSUE 173 (9)IGN	*IMEDIA*__
**172. On the days when you did average activities, how long, in total, did you do average activities per day?**<br>____+____+____+____+____ = __ __ __ minutes per day (888)NSA (999)IGN	*IMIND*__ __ __
**173. In relation to <1 YEAR BEHIND> you consider that your current physical activity is:**<br>(1) Minor<br>(2) Equal - SKIP TO QUESTION 175<br>(3) Largest<br>(9)IGN	*MAFPAS* __
**174. What is the main reason for the change in your practice of physical activity or physical exercise?**<br>____<br>(88)NSA (99)IGN	*MMOTIV*__ __
**175. Since <1 YEAR AGO> have you received guidance for physical activity, sports, physical exercise or gymnastics?**<br>(0) No - SKIP TO QUESTION 182 (1) Yes (9)IGN	*RECORAFANO*__
**NOW LET'S TALK ABOUT THE LAST GUIDANCE RECEIVED FOR THE PRACTICE OF PHYSICAL ACTIVITY**	
**176. Where did you receive this guidance?**<br>(01) Basic Health Unit / Health Post<br>(02) Public outpatient clinic (SUS or college)<br>(03) Ambulatory by health insurance / company plan<br>(04) Private practice / health plan<br>(05) Gym<br>(06) Media (newspaper, magazine, internet, radio, television)<br>(07) Other (88)NSA (99)IGN	*MONREC*__ __
**177. Who guided you?**<br>(01) Doctor<br>(02) Physical Education Teacher<br>(03) Nutritionist<br>(04) Physiotherapist<br>(05) Nurse<br>( ) Other ___________________________ (88)NSA (99)IGN	*MQUEMOR*__ __
**178. What physical activity was advised?**<br>(01) Walking<br>(02) Running<br>(03) Water aerobics<br>(04) Swimming	*MQAFOR*__ __

( ) Other ____ (88)NSA (99)IGN	
**179. Were you instructed on how many times a week <PHYSICAL ACTIVITY> should be done?**<br>(0) No (1)Yes (8)NSA (9)IGN	*MORVEZSEM* __
**180. Were you advised about the time that <PHYSICAL ACTIVITY> should have?**<br>(0) No (1)Yes (8)NSA (9)IGN	*MORTEMP* __
**181. After the orientations received, your physical activity:**<br>(1) Increased<br>(2) Decreased<br>(3) Not changed (8)NSA (9)IGN	*MMUD* __
**182. Since <1 YEAR AGO> have you sought, sought guidance for the practice of physical activity, sports, physical exercises or gymnastics?**<br>No SKIP TO QUESTION 184<br>Yes (9)IGN	*MPROCOR*__
**183. If yes: Where?**<br>(01) Media (newspaper, magazine, television, internet, radio)<br>(02) Health service<br>(03) Gym<br>(04) Work<br>Other________________________ (88)NSA (99)IGN	*MONDPROC*__ __
**184. Do you smoke or have you ever smoked?**<br>(0) No, never smoked - SKIP TO QUESTION 187<br>(1) Yes, smokes (1 or + cigarette (s) per day for more than 1 month)<br>(2) Smoked, but stopped smoking __ __ years __ __ months (9)IGN	*FUMO* __<br>*TPAFA* __ __<br>*TPAFM* __ __
**185. How long have you smoked? (or how long have you smoked)?**<br>__ __ years __ __ months (88)NSA (99)IGN	*TFUMA*__ __<br>*TFUMM*__ __
**186. How many cigarettes do you smoke (or did you) per day?**<br>__ __ cigarettes (88)NSA (99)IGN	*CIGDI*__ __
**187. Did you have any alcoholic beverages in the last 30 days?**<br>(1) Yes (2) No - SKIP TO QUESTION 192 (9)IGN	*BEAL30D* __<br>*CRBBALC*__
**188. Have you ever felt that you should decrease the amount of alcoholic beverages or stop drinking?**<br>(1) Yes (2) No (8)NSA (9)IGN	*CAGE1* __<br>*CRCAG1* __
**189. Do people bother you because they criticize your drinking?**<br>(1) Yes (2) No (8)NSA (9)IGN	*CAGE2* __<br>*CRCAG2* __
**190. Do you feel upset with yourself for the way you usually drink alcohol?**<br>(1) Yes (2) No (8)NSA (9)IGN	*CAGE3* __<br>*CRCAG3* __
**191. Do you drink alcoholic beverages in the morning to reduce nervousness or hangover?**<br>(1) Yes (2) No (8)NSA (9)IGN	*CAGE4* __<br>*CRCAG4* __

NOW LET'S TALK ABOUT FEELINGS

**192. Are you basically satisfied with your life?**	(0)No<br>(1)Yes	ISATIS__
**193. Did you leave many of your interests and activities?**	(0)No<br>(1)Yes	*IINTER*__
**194. Do you feel that your life is empty?**	(0)No<br>(1)Yes	*IVAZIA* __
**195. Do you get bored frequently?**	(0)No<br>(1)Yes	*IABORR* __
**196. Do you feel in a good mood most of the time?**	(0)No<br>(1)Yes	*IHUMOR* __
**197. Are you afraid that something bad will happen to you?**	(0)No<br>(1)Yes	*IMEDO* __
**198. Do you feel happy most of the time??**	(0)No<br>(1)Yes	*IFELIZ* __
**199. Do you feel that your situation has no way out?**	(0)No<br>(1)Yes	*ISAIDA* __
**200. Do you prefer to stay at home rather than go out and do new things?**	(0)No<br>(1)Yes	*IPREFE* __
**201. Do you feel you have more memory problems than most?**	(0)No<br>(1)Yes	*IMEMOR* __

**202. Do you think it's wonderful to be alive?**	(0)No<br>(1)Yes	*IVIVO* __
**203. Do you feel useless in the current circumstances?**	(0)No<br>(1)Yes	*INUTIL* __
**204. Do you feel full of energy?**	(0)No<br>(1)Yes	*IENER* __
**205. Do you think your situation is hopeless?**	(0)No<br>(1)Yes	*ISEMES* __
**206. Do you feel that most people are better than you?**	(0)No<br>(1)Yes	*IMELHO* __
**NOW I WOULD LIKE TO ASK YOU SOME QUESTIONS ABOUT YOUR MEMORY AND REASONING. THERE ARE NO RIGHT OR WRONG ANSWERS AND SOME QUESTIONS MAY SEEM TO BE NON-DIRECT. BUT I WOULD LIKE YOU TO PAY ATTENTION AND ANSWER ALL QUESTIONS AS BEST.**		
**207. What is the <READ ALTERNATIVES> that we are in?**<br>**The day of the week: ______________________**<br>**The day of the month: ________________________**<br>**The month: _______________________________**<br>**The year: ________________________________**<br>**The approximate time: _______:_______**		*DIAS* __<br>*DIAM* __<br>*MÊS* __<br>*ANO* __<br>*HORA* __<br>*OTEMP* __
**208. What is <READ THE ALTERNATIVES> where we are?**<br>**The city () Bagé () other () does not know**<br>**The neighborhood: ____________________ () other () does not know**<br>**The state () RS () other () does not know**<br>**The country () Brazil () other () does not know**<br>**The house / apt part: ___________ () other () does not know**<br><br>IF YOU ARE ON THE STREET, ASK:<br>**Which side of your house are we on? _________() other () does not know**		*CIDADE* __<br>*BAIRRO* __<br>*ESTADO* __<br>*PAIS* __<br>*PEÇA* __<br><br><br>*OESPA* __
**209. I am going to tell you the name of three objects: CARRO, VASO, TIJOLO. Could you repeat for me?**<br>( ) carro ( ) other ( ) don't know<br>( ) vaso ( ) other ( ) don't know<br>( ) tijolo ( ) other ( ) don't know		*CARRO* __<br>*VASO* __<br>*TIJOLO* __
**REPEAT ANSWERS UNTIL THE INDIVIDUAL LEARN THE THREE WORDS (5 ATTEMPTS)**		
**210. Now I'm going to ask you to do some math. How much is it:**<br>**1. 100 - 7: _______**<br>**2. 93 - 7: _______**<br>**3. 86 - 7: _______**<br>**4. 79 - 7: _______**<br>**5. 72 - 7: _______**		*CONTA* __
**211. Could you tell me the name of the 3 objects that I told you before?**<br>( ) carro ( ) other ( ) don't know<br>( ) vaso ( ) other ( ) don't know<br>( ) tijolo ( ) other ( ) don't know		*CARRO1* __<br>*VASO1* __<br>*TIJOLO1* __
**212. What is the name of these objects?** *<SHOW>*<br>A pencil (standard): () pencil () other<br>A wristwatch: () watch () other		*LAPIS* __<br>*RELO* __
**213. I am going to say a sentence: "NOR HERE, NOR THERE, NOR THERE".**<br>**214. Could you repeat?**<br>( ) repeated ( ) did not repeat		*REPET* __
**215. I would like you to do it according to the following instructions: FIRST READ THE 3 INSTRUCTIONS AND ONLY THEN THE INTERVIEWER MUST PERFORM THEM.**<br>**Take this paper with your right hand () fulfilled () did not fulfill**<br>**Fold in half with both hands () fulfilled () not fulfilled**<br>**Put the paper on the floor () did () did not do**		*ETAPA1* __<br>*ETAPA2* __<br>*ETAPA3* __
**216. I am going to show you a written phrase. You will look and without saying anything, you will do what the sentence says. If you wear glasses, please put them on, as it will be easier.**<br>*SHOW THE PHRASE ON THE "CLOSE YOUR EYES" CARD*		*LEI* __

( ) performed task ( ) did not perform task ( ) other	
**217. Could you write a sentence of his choice, any sentence?**<br>*GUIDING THE INTERVIEWEE TO WRITE ON THE FOLLOWING LINE*<br>*(BEFORE DRAWING)*	*FRASE* __
**218. And to finish this part, I would like you to copy this drawing:**<br>*SHOW DRAWING AND GUIDE TO COPY NEXT*	*PRAXIA* __
SPACE ALLOCATED FOR THE PHRASE<br>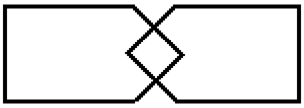	*TOTAL* __ __
**NOW I WILL ASK QUESTIONS ABOUT THE PROPERTY AND INCOME OF THE RESIDENTS OF THE HOUSE.LEMBER, ONCE AGAIN, THAT THE DATA OF THIS STUDY IS CONFIDENTIAL. THEREFORE, BE QUIET (A) TO INFORM WHAT IS ASKED.**	
**219. In your home do you have:**	
**Vacuum Cleaner?** (0) No (1) Yes (9)IGN	*ASP*__
**Washing machine?** (0) No (1) Yes (9)IGN	*LAV*__
**VCR or DVD?** (0) No (1) Yes (9)IGN	*VDVD*__
**Fridge?** (0) No (1) Yes (9)IGN	*GEL*__
**Freezer or duplex refrigerator?** (0) No (1) Yes (9)IGN	*FRDU*__
**Microwave?** (0) No (1) Yes (9)IGN	*MICR*__
**Microcomputer?** (0) No (1) Yes (9)IGN	*MICROCOMP*__
**Phone?** (0) No (1) Yes (9)IGN	*TELFIX*__
**220. In your home, do you have ...? How many?**	
**Rádio** (0) (1) (2) (3) (4+) (9)IGN	*RAD*__
**Black and White television** (0) (1) (2) (3) (4+) (9)IGN	*TVPB*__
**Color television** (0) (1) (2) (3) (4+) (9)IGN	*TVCOL*__
**Car***(private use only)* (0) (1) (2) (3) (4+) (9)IGN	*AUT*__
**221. Do you have a monthly housekeeper or domestic servant at home? If so, how many?**<br>(0) No (1) Yes, how many? __	*EMPDOM*__<br>*EMPDOMQT*__
**222. How many people live in this house?**<br>___ ___ persons<br>(99)IGN	*QTMOCASA*__ __
**223. How many pieces are used for sleeping?**<br>___ ___ pieces<br>(99)IGN	*QTPECDOR*__ __
**224. How many bathrooms are there in the house? (consider only those that have a toilet plus shower or bathtub.**<br>___ ___ bathrooms<br>(99)IGN	*QTBANCA* __ __
**225. What is the education level of the person who has the highest income at home?**<br>(1) none or up to 3rd grade (incomplete primary)<br>(2) 4th grade (complete primary) or 1st grade (junior high) incomplete<br>(3) Complete high school or incomplete high school<br>(4) Complete high school or incomplete higher education<br>(5) complete higher education<br>(9)IGN	*ESCPESMREND*__

**226. How much did the people living here last month earn, including work and retirement?**<br>Person 1: R$ __ __ __ __ __ per month<br>Person 2: R$ __ __ __ __ __ per month<br>Person 3: R$ __ __ __ __ __ per month<br>Person 4: R$ __ __ __ __ __ per month<br>Person 5: R$ __ __ __ __ __ per month<br>(00000) No income (88888)NSA (99999)IGN	*BRF1*__ __ __ __ __<br>*BRF2*__ __ __ __ __<br>*BRF3*__ __ __ __ __<br>*BRF4*__ __ __ __ __<br>*BRF5*__ __ __ __ __
**227. Does the family have another source of income, for example, rent, pension or other that was not mentioned above?**<br>(0) No (1) Yes - **How much? R$ __ __ __ __ __ per month**<br>(99999)IGN	*OUTFREN*__<br>*OUTQT*__ __ __ __ __
**228. Do you have any health plan or health insurance (such as UNIMED, IPE, FUSEX, Union)?**<br>(0) No<br>(1) Yes. Which?____________________________<br>(9)IGN	*QUERESP*__
TO COMPLETE THE INTERVIEWER:<br>The questionnaire was answered:<br>(1) All by the elderly, without help<br>(2) All by the elderly, with help<br>(3) Some answers were given by someone else<br>(4) Most of the answers were given by someone else<br>(5) All answers were given by someone else	
End time of interview: __ __ : __ __ hs	

CLOSE THE QUESTIONNAIRE AND THANK THE COLLABORATION
